# Case of Gun Shot Wound of the Superior Extremity

**Published:** 1859-04

**Authors:** 


*Case of Gun Shot Wound of the Superior Extremity. Post-mortem appearances in a case of disease of the Abdominal Lymphatic Glands.*

Editor of Buffalo Medical Journal.

SIR: If you think the following cases worthy a place in the Journal, they are at your dispcsal:

C. A., æt. 20, in good health, on the 28th Sept., 1858, was standing on a large fallen tree, beside which he had placed his loaded rifle; stooping forward, he grasped the gun near the muzzle, his forearm being flexed or bent upon the arm at an angle of 45°; in bringing the arm upward, it was discharged. The powder burned and blackened the palm and wrist. The ball, (size, 80 to the lb.) cotton patch, and double pieces of cotton shirt, entered the palmar side of the forearm (making an opening an inch in diameter) one and a-half inches anterior to the elbow-joint, striking the humerus between the condyles. The fracture was longitudinal, making two triangular fragments, either four inches in length, apex towards the shoulder; the triangular portion between these fragments, apex at the elbow; was in three pieces. The ball passed (we traced the passage clearly by a blue streak and bubbles of air) externally to the humeral artery, near the external wall of the axilla, and lodged beneath the skin, near the inferior angle of the scapula.

The forearm was pale, cold, and pulseless; the pulsation of the humeral artery was distinct from the arm-pit to the small central fragments; then upon the opposite side of the arm, was a full pulse. Appearing to emerge from between the fragments, it continued toward the elbow, two and a-half inches, and disappearing between the fragments. External hæmorrhage slight; considerable blood extravasated, the arm very crooked and deformed, and supposing the humeral artery torn off, it appeared a clear case for amputation.

The teachings of Prof. F. H. Hamilton, on conservative surgery, (in 1847, I had the benefit of Prof. Hamilton's lectures on surgery in the Geneva Medical College,) and the case of the child whose arm was so mashed and torn by a railroad car, reported last year in the Buffalo Medical Journal, determined an attempt to save the arm at a risk to the patient's life and the doctor's reputation.

*Treatment.* After a fruitless search for the cotton cloth, ½ gr. morphine was administered, the forearm flexed and bandaged from shoulder to finger ends, (the object of bandage to favor oozing from wound and absorption of extravasated blood) and placed in an angular box. At the end of the second day, the object of the bandage being attained, it was removed, and cold water applied by two small siphons. Ep. salts repeated daily; diet, watei gruel. On the fourth day, the patient had chills, increased swelling and pain in the arm; headache, and the pulse at 90, and tense.

Gave five drops fluid ext. veratrum, to be repeated every four hours until the pulse was at 60.

Fifth day. Pain less; swelling increasing; pulse 60.

Diminished the dose of veratrum to three drops.

At night I was summoned to the patient, who had severe pain in the head and arm; complained of "burning up;" pulse 95.

Increased veratrum to five drop doses.

Sixth day. Pulse 60.

Continued veratrum, salts, gruel, and siphons, till the tenth day, when the swelling had much diminished.

We adjusted the fragments, bandaged the limb, and applied a pasteboard and paste splint over either condyle. This dressing was allowed to remain undisturbed till the seventeenth day, when the splints were removed and the arm replaced in the box.

Fearing anchylosis, we removed the splints thus early and commenced moving the forearm, while an assistant grasped tightly with both hands the humerus. At first the forearm could not be extended, as the olecranon process came in contact with the internal condyle; but by continued effort, we succeeded in moving it.

At present, March 15th, 1859, the motions of the forearm and arm are natural and easy. The thumb, index and middle fingers, are sparingly supplied with blood, having less heat and strength than the other parts of the hand. There is no perceptible pulse in the forearm, and we find the same two and a-half inches of artery, (appearing as large as the humeral artery,) on the opposite or outside of the arm. The wound has been healed three months. We have seen nothing of the pieces of cotton.

*Queries.* Did the pieces of cotton cloth enter the wound? Was the humeral artery divided? (Remember the member is useful and almost natural.) How can we account for the two and a-half inches of pulsation as distinct and large as the humeral artery?

To fill this sheet, I give the *post-mortem* examination of W. H., æt. 5 years, who, at the age of four months, had enlargement of the abdomen.

The diagnosis, (by Dr. Briggs, of Auburn,) at that time, was tabes mesenterica.

Twelve hours after death. We found twelve pounds of serous fluid in the abdominal cavity; the lesser and greater omentum, a mass of enlarged glands, varying in size from that of a large pea to the size of a tomato, one and a-half inches in diameter, resembling in form and color a ripe tomato. Then appeared a tumor filling the entire pelvic, and a great part of the abdominal cavities. The stomach and small intestines were in the hypochondriac region, healthy, but much contracted. The sigmoid flexure and rectum were firmly bound and attached to the tumor, effectually preventing any but liquid passages. The bladder, capable of containing only two fluid drachms, was adhered and bound by fibrous bands to the tumor. Three inches of either ureter were imbedded in the tumor, and so contracted as to prevent the passage of urine as fast as secreted. The remaining six inches of the ureter were dilated to an inch and a-half in diameter at the pelvis of the kidney, and one inch at the tumor: the ureters being capable of containing as much as a common sized bladder. The tumor, a part of the colon, the rectum, ureters, and bladder, were tied and removed, then separated. The tumor measured twenty-four inches in circumference, had a thick fibrous sac, enclosing what appeared like chalk or bone deposits a quarter of an inch in thickness, with intermediate glandular layers an inch in thickness.

We now removed the remaining viscera of the abdomen. The diaphragm was covered by contiguous rows, running from sternum to spine, of glands of uniform shape, size and color, of a flattened tomato—one diameter $\frac{3}{4}$, and the other 1 inch. There were similar rows from the sacrum to the diaphragm, covering the posterior walls of the abdomen and the lateral walls of the right and left hypochondriac, lumbar, and iliac regions. Innumerable glands of the size of peas, and fibrous, were seen in the peritoneum covering the liver, stomach, and intestines.

Yours respectfully,

MARTVILLE, Cayuga Co., N. Y. WM. S. KYLE, M. D.

---

Dr. THOMAS WATSON has been appointed Physician Extraordinary to Her Majesty, Queen Victoria, in place of the lamented Dr. Richard Bright. Dr. Watson is well known to the profession for his high character and distinguished attainments, and as the author of the "Principles and Practice of Physic."—*Boston Med. & Surg. Journal.*

